# Do not expect an endoluminal complete response to identify a pathologic complete response in rectal cancer!

**DOI:** 10.1016/j.clinsp.2025.100587

**Published:** 2025-02-04

**Authors:** Alexandre Gheller, Guilherme Inácio Bertoldo de Melo e Patriarca da Silva Neiva, Mario Nóbrega de Araújo Neto, Fernando Gonçalves Lyrio, Dunya Bachour Basílio, Marília Cristina Roda da Costa, Douglas Vieira Mourão, Paulo Gonçalves de Oliveira, João Batista de Sousa

**Affiliations:** a Colorectal Surgery Department, Hospital de Base do Distrito Federal, Brasília, DF, Brazil; b Abdominal and Pelvic Surgery Department, Instituto Nacional de Câncer (INCA), Rio de Janeiro, RJ, Brazil; c Pathology Department, Hospital de Base do Distrito Federal, Brasília, DF, Brazil; d Division of Colorectal Surgery, Universidade de Brasília (UnB), Brasília, DF, Brazil

**Keywords:** Rectal câncer, Pathologic complete response, Endoluminal complete response, Chemoradiotherapy

## Abstract

**Purpose:**

To examine the relationship between endoluminal and pathologic complete response after chemoradiotherapy for rectal cancer and identify predictors of a pathologic complete response.

**Method:**

The anatomic pathology reports of 102 consecutive patients with rectal cancer who underwent neoadjuvant chemoradiotherapy followed by proctectomy between 2013 and 2017 were reviewed for the presence or absence of endoluminal complete response. The presence of endoluminal complete response was compared with the anatomopathological stage. The residual lesion area was compared with the final pathologic stage to identify predictors of complete response.

**Results:**

Of 102 patients, 20 (19.6 %) achieved a pathologic complete response (ypT0N0). Of these, 9 (45 %) did not achieve an endoluminal complete response. The presence of endoluminal complete response had a sensitivity of 55.00 %, specificity of 96.34 %, and accuracy of 88.24 % to identify ypT0N0. The presence of endoluminal complete response, residual lesion area ≤ 4 cm^2^, and tumor located in the mid-rectum were associated with pathologic complete response (ypT0N0).

**Conclusion:**

Almost half of the patients who had a pathologic complete response did not achieve an endoluminal complete response. Tumors located in the mid-rectum with a residual size of ≤ 4 cm^2^ and the presence of endoluminal complete response were significantly associated with the achievement of ypT0N0.

## Highlights

• Correlation between pathologic complete response and endoluminal complete response.
• Half of pathologic complete responses do not achieve an endoluminal complete response.
• Gross findings do not perfectly reflect microscopic findings.

## Introduction

The use of neoadjuvant Chemoradiotherapy (CRT) can lead to a reduction in tumor volume (downsizing) and stage (downstaging), and in up to one-third of cases no residual tumor cells are detected, the so-called pathologic Complete Response (pCR; ypT0N0). The observation of this phenomenon has allowed the adoption of organ preservation strategies, especially watch-and-wait management and local excision.^1^

The difficulty in implementing the watch-and-wait strategy lies in identifying which patients have achieved ypT0N0. In an attempt to overcome this difficulty, an international group of experts published a series of endoluminal morphologic criteria that are closely associated with the achievement of ypT0N0.^2^ The criteria include the absence of a visible or palpable tumor on clinical-endoscopic examination and the presence of areas of pallor, scarring, or telangiectasia where the tumor was located. These criteria have become the definition of the endoluminal component of a clinical Complete Response (cCR).

Smith et al.^3^ reviewed the pathology reports of patients with rectal cancer undergoing neoadjuvant CRT to compare the gross endoluminal appearance of the resected specimens with pathologic T and N stages. They reported that the application of the endoluminal morphologic criteria of cCR, hereafter referred to as endoluminal Complete Response (eCR), had high specificity (97 %) and low sensitivity (26 %) to identify patients with ypT0N0.^3^

The most commonly used criterion for identifying likely complete responders is the occurrence of eCR. Nevertheless, the association between ypT0N0 and eCR remains inconsistent across studies. Therefore, the present study aimed to examine the relationship between eCR and pCR (ypT0N0) after neoadjuvant CRT for rectal cancer and to identify predictors of ypT0N0.

## Materials and methods

A retrospective cohort study included 124 patients with adenocarcinoma of the mid and lower rectum, <10 cm from the anal verge, between 2013 and 2017. Patients were identified from an electronic database of medical records, prospectively updated, containing cases operated on at the University Hospital of Brasilia and at the Federal District Base Hospital. The sample was obtained consecutively in the units of the two-member centers. The study was approved by the Research Ethics Committee of Brasilia University (UnB) Medical School and followed the tenets of the Declaration of Helsinki.

Baseline assessment and staging included a thorough proctoscopic examination, colonoscopy, dedicated pelvic Magnetic Resonance Imaging (MRI) for rectal cancer, and computed tomography of the chest and abdomen. Restaging was performed between 8 and 14 weeks after the end of neoadjuvant CRT and consisted of a thorough proctoscopic examination, flexible rectosigmoidoscopy, and pelvic MRI. Surgery was performed up to 7 weeks after restaging.

All patients analyzed underwent similar staging and anatomopathological analysis. Exclusion criteria were failure to complete the proposed neoadjuvant CRT, stage IV disease, early postoperative death, palliative resections, and previous exposure to CRT for another neoplasm. Patients with rectal adenocarcinoma over 18 years of age, stage II and III, and complete medical records were included.

All eligible patients received 5-fluorouracil-based chemotherapy with leucovorin or oral capecitabine concomitant with radiation at weeks 1 and 5. Conformal external radiotherapy was delivered to a total dose of 5040 cGy in 30 fractions. Surgical procedures involved low anterior and abdominoperineal resections. Surgical specimens were subjected to pathologic analysis and staged according to the American Joint Committee on Cancer guidelines. Pathology reports were reviewed and individually searched by 2 authors (A.G. and M.N.A.N.) for points of relevance.

The variables of interest were as follows: sex; age 〈 60-years or ≥ 60-years; tumor located in the lower rectum or in the mid rectum; clinical stage II or III; low anterior resection or abdominoperineal resection; presence or absence of lesion in the specimen; well or poor/moderate tumor differentiation; interval between neoadjuvant CRT and surgery ≤ 13-weeks or 〉 13-weeks; presence or absence of perineural invasion; presence or absence of lymphovascular invasion; and residual lesion area ≤ 4 cm^2^ or > 4 cm^2^.

**Fig. 1:**
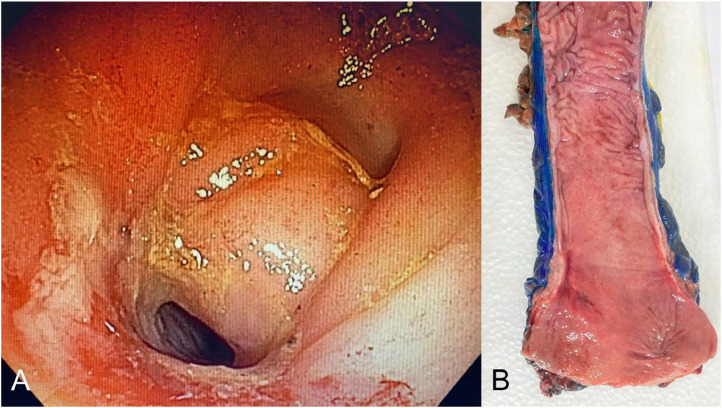
Endoluminal Complete Response (eCR) without pathologic Complete Response (pCR). (A) Endoscopic view and (B) resected specimen.

**Fig. 2:**
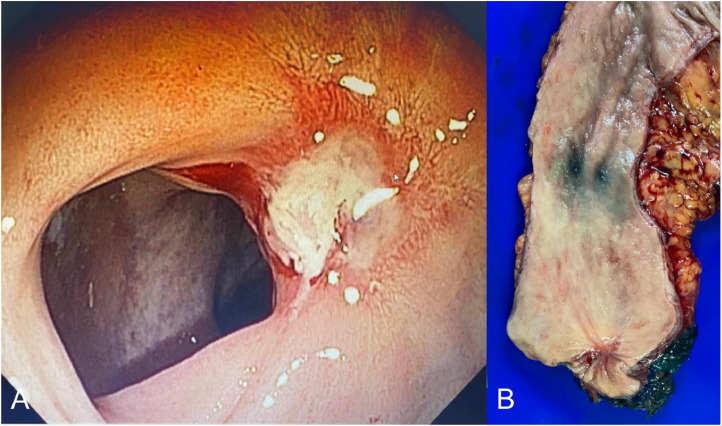
Pathologic Complete Response (pCR) without endoluminal Complete Response (eCR). (A) Endoscopic view and (B) resected specimen.

The primary outcome was the presence or absence of an endoluminal component of a cCR. Any endoluminal finding other than mucosal pallor, scarring, or telangiectasia was considered the absence of eCR, and the residual lesion area was immediately calculated, including the calculation of area size (cm^2^) and the ypT and ypN stage for each tumor, and achievement of ypT0N0 (Fig. 1, Fig. 2).

### Statistical analysis

The results were described by descriptive statistics and reported as means and Standard Deviation (SD). Bivariate and multiple regression analyses were performed for the characteristics of the study variables associated with ypT0N0 and ypT0N0 without eCR, using the prevalence ratio as an effect measure. For both, frequencies and prevalence ratios of the variables of interest were calculated. Multiple Poisson regression with robust variance was performed using the achievement of ypT0N0 and ypT0N0 without eCR as dependent variables. Those variables with a p-value < 0.25 were included in the multiple Poisson regression analysis. After adjustments, only those variables with a p-value < 0.05 remained in the final model. Multicollinearity among the independent variables was assessed, and a tolerance value < 0.40 indicated a multicollinearity problem. A p-value < 0.05 was considered significant. All analyses were performed using SAS 9.4 (SAS Institute, Cary, NC, USA).^4^, ^5^, ^6^

## Results

Of 124 patients initially identified, 22 were excluded: 9 patients operated on in other facilities, 7 patients failing to complete the proposed CRT regimen, 5 patients with stage IV disease, and 1 patient with previous exposure to CRT for another neoplasm.

A total of 102 patients met the inclusion criteria and had complete pathologic data, including detailed gross mucosal descriptions. Sixty-one were men and 41 were women, with a median age of 66 years (range, 36–85). Preoperative staging data were available for all 102 patients: clinical stage II, *n* = 51 (50.0 %); clinical stage III, *n* = 51 (50.0 %). Overall, 20 patients were cT2, 68 were cT3, and 14 were cT4 on preoperative staging; 51 of 102 (50.0 %) patients were assessed as having positive nodes. The median interval between the end of neoadjuvant CRT and restaging was 9-weeks. All patients were treated with neoadjuvant CRT with a dose of 5040 cGy. Surgical management involved 65 (63.7 %) low anterior resections and 37 (36.2 %) abdominoperineal resections (Table 1).

**Table 1:** Characteristics of the study population (*n* = 102).

**Variable**	**Values**
Sex, n (%)	
Male	61 (59.8)
Female	41 (40.2)
Age, n (%)	
< 60 years	39 (38.3)
≥ 60 years	63 (61.7)
TNM stage, n (%)	
II	51 (50.0)
III	51 (50.0)
Tumor stage, n (%)	
T2	20 (19.6)
T3	68 (66.6)
T4	14 (13.7)
Tumor site, n (%)	
Mid rectum	48 (47.0)
Lower rectum	54 (53.0)
Surgical technique, n (%)	
Abdominoperineal resection	37 (36.2)
Low anterior resection	65 (63.7)
Interval between neoadjuvant CRT and surgery, n (%)	
≤ 13 weeks	57 (55.8)
> 13 weeks	45 (44.2)
Tumor differentiation, n (%)	
Well	27 (26.4)
Moderate or poor	75 (73.5)
Lymphovascular invasion, n (%)	
Presence	29 (28.4)
Absence	73 (71.6)
Perineural invasion, n (%)	
Presence	26 (25.4)
Absence	76 (74.6)
Endoluminal residual lesion, n (%)	
Presence	87 (85.2)
Absence	15 (14.8)
Residual lesion area, n (%)	
≤ 4 cm^2^	42 (41.1)
> 4 cm^2^	60 (58.9)
Pathologic staging, n (%)	
ypT0N0	20 (19.6)
I	33 (32.3)
II	19 (18.6)
III	30 (29.4)
T pathologic staging, n (%)	
0	20 (19.6)
1	07 (5.8)
2	30 (29.4)
3	38 (37.2)
4	07 (6.8 %)

CRT, Chemoradiotherapy.

**Fig. 3:**
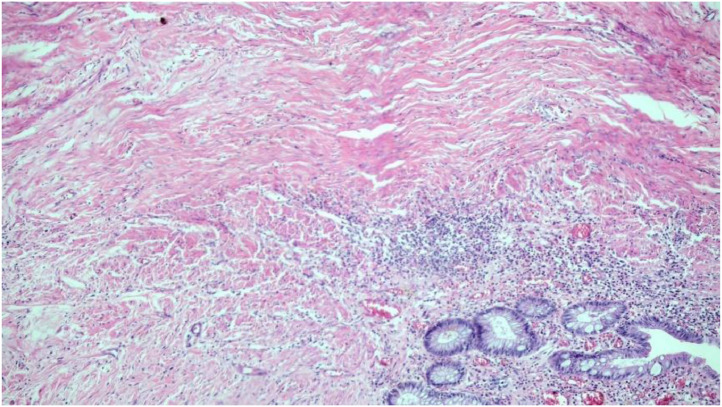
Complete tumor regression with cicatricial fibrosis.

**Fig. 4:**
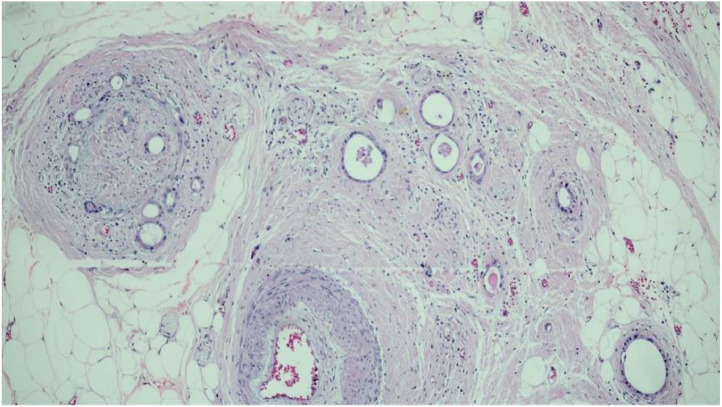
Residual neoplasm present in the perirectal adipose tissue, with neural invasion.

The anatomopathologic results were divided into 2 groups as follows: the relationship between ypT0N0 and eCR; and predictors of ypT0N0 (Table 2; Fig. 3, Fig. 4).

**Table 2:** Results of endoluminal complete response in 20 patients with pCR and 82 patients without pCR.

**pCR**	**Endoluminal complete response**	
	**Positive**	**Negative**
Positive	11 (55.00 %)	9 (45.00 %)
Negative	3 (3.66 %)	79 (96.34 %)

pCR, pathologic Complete Response.

The distribution of study variables according to crude prevalence ratios and adjusted by Poisson regression with robust variance, and their respective 95 % Confidence Intervals are shown in Table 3.

**Table 3:** Predictors of ypT0.

**Variables**	**Crude PR**		**Adjusted PR**^a^	
	**PR (95 % CI)**	**p‒value**	**PR (95 % CI)**	**p-value**
Sex		0.3110		‒
Female	1	‒	‒	‒
Male	1.57 (0.66; 3.75)	0.3110	‒	‒
Age		0.4863		‒
< 60-years	1.32 (0.60; 2.90)	0.4863		‒
≥ 60-years	1	‒	‒	‒
Tumor site		0.0828		0.0273
Mid rectum	2.09 (0.91; 4.80)	0.0828	2.06 (1.08; 3.93)	0.0273
Lower rectum	1	‒	1	‒
Preoperative stage		0.1452		‒
II	1.86 (0.81; 4.27)	0.1452	‒	‒
III	1	‒	‒	‒
Surgery type		0.8950		‒
APR	1	‒	‒	‒
LAR	1.06 (0.46; 2.41)	0.8950	‒	‒
Residual lesion		<0.0001		0.0002
No	7.09 (3.56; 14.30)	<0.0001	4.00 (1.95; 8.21)	0.0002
Yes	1	‒	1	‒
Residual lesion area		0.0035		0.0362
≤ 4 cm^2^	5.67 (1.77; 18.16)	0.0035	3.97 (1.09; 14.46)	0.0362
> 4 cm^2^	1	‒	1	‒
Tumor differentiation		0.4763		‒
Moderate or poor	1.44 (0.53; 3.93)	0.4763	‒	‒
Well	1	‒	‒	‒
Interval between neoadjuvant CRT and surgery		0.1701		‒
≤ 13 weeks	1.84 (0.77; 4.41)	0.1701	‒	‒
> 13 weeks	1	‒		‒
Perineural invasion		0.0614		‒
Absent	6.50 (0.91; 46.19)	0.0614	‒	‒
Present	1	‒	‒	‒

PR, Prevalence Ratio; CI, Confidence Interval; APR, Abdominoperineal Resection; LAR, Low Anterior Resection; CRT, Chemoradiotherapy.

a Adjusted for tumor site, residual lesion, and residual lesion area.

### Relationship between ypT0N0 and eCR

Twenty patients (19.6 %) achieved ypT0N0. Of these, 9 (45.0 %) did not achieve eCR. Of the 14 patients with an eCR, 3 did not achieve ypT0N0; 2 of them with T2N0 and 1 with TisN0. Therefore, the presence of eCR had a sensitivity of 55.00 %, specificity of 96.34 %, and accuracy of 88.24 % to identify ypT0N0. The positive predictive value was 90.21 and the negative predictive value was 77.74.

### Predictors of ypT0N0

Only mid-rectum tumor site, absence of residual lesion, and residual lesion area ≤4 cm^2^ were significantly associated with the presence of ypT0N0.

## Discussion

The use of endoluminal morphologic criteria, known to be associated with the achievement of ypT0N0, is still the most commonly used method for selecting patients for less invasive strategies in rectal cancer.^2^ The present data shows that the achievement of eCR is closely related to the achievement of ypT0N0, demonstrating high specificity (96.34 %) and a high chance (90.21 %) of achieving ypT0N0 in the presence of eCR.

In contrast, Hiotis et al.,^7^ in a retrospective analysis of 488 patients with rectal cancer undergoing neoadjuvant CRT, reported that only 25 % of the patients who achieved cCR also achieved ypT0N0. Their findings probably reflect the wide variation in the definition of cCR used until 2011, when an international group of experts clearly characterized the endoluminal component of Ccr.^2^

In the present study, the use of the eCR criteria allowed for the identification of only half of the patients with ypT0N0, indicating low sensitivity (55 %). These findings are consistent with recent reports of high specificity (97 %) and low sensitivity (26 %) of the application of the endoluminal morphologic criteria of cCR in identifying patients with ypT0N0.^2^^,^^8^

Safatle-Ribeiro et al.,^9^ in a recent prospective study of 47 patients with mid-distal rectal cancer (T3-T4 or N+) treated with neoadjuvant CRT, obtained 4 cases of pCR, 3 of which did not achieve eCR. The present findings are consistent with these data, where a substantial number of pCR cases will not achieve an eCR.

These results suggest that, if the authors were to implement a watch-and-wait strategy, half of the patients with ypT0N0 would have undergone radical surgery unnecessarily. In order to optimize the identification of patients with ypT0N0, the authors searched for clinicopathologic factors associated with this phenomenon, such as the interval between the end of neoadjuvant CRT and surgery > 13 weeks and lower pre-neoadjuvant CRT T stage, and both were associated with higher rates of pCR.^10^^,^^11^

Tumors located in the mid-rectum with a residual size of ≤4 cm^2^ and the presence of eCR were significantly associated with the achievement of ypT0N0. Although mid rectum tumor site was associated with a greater chance of achieving ypT0N0 in our study, previous studies have shown different results. Das et al.^12^ searched for predictors of ypT0N0 in 562 patients with rectal cancer who received preoperative CRT and identified that low rectal tumors were associated with an increased chance of achieving ypT0N0; however, they were unable to justify this finding. It has been hypothesized that tumors closer to the anal verge would be less mobile and, consequently, more easily exposed to radiotherapy.^12^ This hypothesis does not seem to be a viable explanation, since the mid-rectum is located intrapelvically and, logically, lacks mobility. In addition, exposure to radiotherapy, at least in theory, would have the same intensity in both the low and mid rectum. It is known that low rectal tumors are associated with increased local recurrence, and this may be related to surgical technique difficulty or to the more aggressive biological behavior of tumors at this site.^13^ The latter might actually explain the finding of a greater chance of achieving ypT0 in the mid-rectum than in the lower rectum.

An association of the size of residual lesions in the rectum with the achievement of ypT0N0 has been reported by some research groups.^3^^,^^8^^,^^14^ Smith et al.^3^^,^^8^ reported that a residual lesion size of < 1 cm in its greatest diameter was significantly associated with ypT0 status. These results are similar to ours; they only differ in that the authors used residual lesion area (in cm^2^) rather than the largest diameter (in cm) as a variable.

The present study has potential limitations that need to be addressed. The identification of predictive factors for ypT0N0 and ypT0N0 without eCR was based on a retrospective analysis of data from a prospectively updated database encompassing 2 large departments of colorectal surgery in the Midwest of Brazil but the retrospective nature of data collection limits the generalizability of these findings. Application of these factors to a population requires external validation by other institutions. However, all patients in this series had the same initial staging examinations, were treated with the same neoadjuvant CRT, and were operated on for total mesorectal excision by surgeons with extensive experience in the technique. In addition, a prospective study of patients with rectal cancer undergoing CRT and radical surgery after achieving cCR seems difficult to perform, mainly because achieving a cCR is not common. Also, exposing patients to mutilating surgery while being aware that they have a high chance of achieving ypT0N0 would be considered unethical.

A potential source of criticism is the possible difference between gross endoluminal pathologic analysis and gross endoluminal analysis in a clinical setting, using endoscopic images. The authors believe that the size of a residual endoluminal lesion observed in anatomopathological examination, as in the present study, may show a correlation with that observed *in vivo*. Although the action of formaldehyde may reduce the size of residual endoluminal lesions, this does not detract from the present findings. On the contrary, it will only increase the specificity of residual lesion area calculation in identifying ypT0N0.

## Conclusion

Almost half of the patients who achieved a pCR did not achieve an eCR. Tumors located in the mid-rectum with a residual size of ≤4 cm^2^ and the presence of eCR were significantly associated with the achievement of ypT0N0.

## Ethics approval

The study was approved by the Research Ethics Committee of Universidade de Brasilia (UnB) Medical School and followed the tenets of the Declaration of Helsinki.

## Consent to participate

Not applicable.

## Availability of data and material

All relevant data are within the paper.

## Authors’ contributions

Alexandre Gheller: Conceptualization, data curation, formal analysis, investigation, methodology, project administration, resources, supervision, validation, writing-original draft preparation; writing-review & editing.

Guilherme Inácio Bertoldo de Melo e Patriarca da Silva Neiva: Software, visualization.

Mario nóbrega de araújo neto: conceptualization, data curation, formal analysis, investigation, methodology, writing-original draft preparation.

Fernando Gonçalves Lyrio: Software, visualization.

Dunya Bachour Basílio: Software, visualization.

Marília Cristina Roda da Costa: Software, visualization.

Douglas Vieira Mourão: Software, visualization.

Paulo Gonçalves de Oliveira: Formal analysis, validation, writing-original draft preparation, writing-review & editing.

João Batista de Sousa: Conceptualization, data curation, formal analysis, investigation, methodology, project administration, resources, supervision, validation, writing-original draft preparation, writing-review & editing.

## Funding

This research did not receive any specific grant from funding agencies in the public, commercial, or not-for-profit sectors.

## Declaration of competing interest

The authors declare no conflicts of interest.
